# Severe hypercalcemia due to metastatic pancreatic neuroendocrine tumor: a case report

**DOI:** 10.1186/s13256-023-04042-5

**Published:** 2023-09-06

**Authors:** Aram Behdadnia, Marjan Jeddi

**Affiliations:** Endocrinology and Metabolism Research Center, Nemazee Hospital, Shiraz University of Medical Sciences, Shiraz, 71345-1414 Iran

**Keywords:** Hypercalcemia, Neuroendocrine tumor, Pancreas, PTHrP

## Abstract

**Background:**

Hypercalcemia of malignancy, as a paraneoplastic syndrome, is the most common metabolic disorder that accounts for 30% of malignancies and usually has a poor prognosis. Neuroendocrine tumors are uncommon and arise from neuroendocrine cells throughout the body. Actually, paraneoplastic hypercalcemia in neuroendocrine tumors is unusual and mostly associated with parathyroid hormone-related protein (PTHrP) secretion.

**Case presentation:**

We report a 51-year-old Iranian man who presented with nausea, vomiting, and significant weight loss for 1 month. Laboratory data revealed calcium of 26 mg/dl, accompanied by low level of PTH. Octreotide scan revealed a large donut-shaped octreotide avid lesion in the epigastric region at the right side of the mid-abdomen, with multiple varying size foci of abnormally increased radiotracer uptake in the epigastric region and both lobes of the liver. Endoscopic ultrasonography demonstrated a large heterogeneous mass lesion with irregular outline and good demarcation in the body of the pancreas with diffuse foci of calcification. Percutaneous biopsy of the liver mass demonstrated a well-differentiated neuroendocrine tumor (low grade) confirmed by immunohistochemistry with strongly positive chromogranin and synaptophysin stain. Hypercalcemia was treated with hydration, few sessions of hemodialysis, calcitonin, and denosumab injection. However, the patient developed symptomatic hypocalcemia. Oncology consultation led to prescription of long-acting octreotide 30 mg monthly and everolimus daily.

**Conclusion:**

Pancreatic neuroendocrine tumor could lead to malignant hypercalcemia; secretion of PTHrP is the most common cause, and signs and symptoms are usually milder than paraneoplastic syndrome due to hematologic and solid tumor. Generally, survival is better; however, its treatment is challenging, and primary debulking surgery is often required. A team approach to management is important at all points.

## Background

Paraneoplastic syndromes (PNSs) are various clinical states affecting different systems, which represent with clinical symptom and signs associated with malignancies. PNS is the result of tumor-mediated bioactive substance or immune-mediated production that does not connect with the origin of the tumor or certain organ. PNS could happen before, simultaneously, or after the diagnosis of tumor and could affect the tumor management. Also, PNS could affect the quality of life and prognosis [1, 2].

Hypercalcemia of malignancy as a PNS is the most common metabolic disorder that affects up to 30% of advanced malignancies and usually has a poor prognosis [3, 4]. It occurs through four mechanisms: (1) parathyroid hormone-related protein (PTHrP) secretion, (2) osteolytic metastases, (3) 1,25-dihydroxyvitamin D overproduction, and (4) ectopic hyperparathyroidism [5].

Clinical presentation of hypercalcemia of malignancy resembles benign hypercalcemia, which consists of gastrointestinal, cardiovascular, and neurologic signs and symptoms. Cardiovascular and neurologic complaints occur especially with a calcium level higher than 14 mg/dl. On the other hand, it may have a gradual course and nonspecific presentation, causing delay in diagnosis [6].

PTHrP, like Parathyroid Hormone (PTH), leads to bone resorption via the active nuclear factor-B RANK/RANK ligand system. Although PTHrP can increase the renal calcium reabsorption, only PTH could lead to increased intestinal calcium absorption through 1,25-dihydroxyvitamin D synthesis [7]. Hypercalcemia due to 1,25-dihydroxyvitamin D occurs from increased calcium resorption of the bone and intestinal tract [7]. Early recognition and treatment is vital as hypercalcemia of malignancy may be life threatening.

Neuroendocrine tumors (NETs) are uncommon and arise from the neuroendocrine cells that are distributed throughout the body and secrete active peptide and amine hormones that can manifest with variable clinical syndrome [8].

Different clinical presentations, various aggressive courses, and different hormone secretions lead to complicated tumor diagnosis and management [9].

Paraneoplastic hypercalcemia in NET is unusual [10]. However, it usually occurs with gastroenteropancreatic NETs, commonly pancreatic NETs (PNET) [11], and is mostly associated with PTHrP secretion, so it is described as humoral hypercalcemia of malignancy (HHM) [5].

There are some case reports of NETs with hypercalcemia with different presentations related to various stages and grades of the tumor and different managements [12]. While a known overt malignancy commonly results in HHM, occasionally it could be the first manifestation of malignancy, so it is worth considering occult malignancy in this regard.

Here, we report a rare case of a 51-year-old man with resistant malignant paraneoplastic hypercalcemia due to an undiagnosed metastatic pancreatic neuroendocrine tumor, which lead to symptomatic hypercalcemia.

## Case presentation

A 51-year-old Iranian married man in Fars province in the south of Iran presented with complaints of nausea, generalized headache, vomiting, malaise, and significant weight loss of about 13 kg in the past 1 month. He had a weight of 65 kg, height of 175 cm, blood pressure of 120/80 mmHg, pulse rate of 68 beats per minute, and blood glucose level of 143 mg/dl. General examination was unremarkable, except for fullness in the right upper quadrant of the abdomen.

A review of his medical history showed that he had diabetes mellitus for 1 year. Social history of the patient was negative for alcohol consumption and cigarette smoking. His family history included diabetes mellitus in his mother, and his drug history consisted of metformin 500 mg daily. For better evaluation, the patient was admitted, and the subsequent laboratory results revealed severe hypercalcemia of more than 15 mg/dl and phosphorous 4.5 mg/dl. He had abnormally low PTH level of 4.37 pg/ml, as well as a low 25 (OH) vitamin D level of 14 nmol/l. Blood urea nitrogen (BUN) was 47 mg/dl, creatinine was 4.12 mg/dl, and potassium was 4.1 meq/l (Table 1).

**Table 1:** Laboratory data of the pati﻿ent

	Result	Reference range
White blood cell count (10^3^/µl)	9.3	4–10
Hemoglobin (g/dl)	11.4	14–18
Platelets count (10^3^/µl)	163	150–450
Blood sugar (mg/dl)	143	
Calcium (mg/dl)	> 15	8.6–10.3
Phosphorus (mg/dl)	4.5	2.3–3.7
Albumin (g/dl)	4	3.5–5.2
Blood urea nitrogen (mg/dl)	47	8–20
Creatinine (Cr) (mg/dl)	4.2	0.8–1
Sodium (Na) (mEq/l)	136	136–145
Potassium (K)(mEq/l)	4.1	3.5–5.5
Alanine transferase (ALT) (U/L)	21	< 40
Aspartate transferase (AST) (U/L)	16	< 45
ALP (U/L)	545	64–306
PTH (pg/ml)	4.37	15–65
25OHD3 (nmol/l)	14.9	
Total bilirubin (mg/dl)	0.6	0.1–1.2
Direct bilirubin (mg/dl)	0.30	< 0.3
PH PCO_2_ (mmHg) HCO_3_ (mmol/l)	7.35 35 18	
PSA (ng/ml)	0.65	0–4

*ALP* Alkaline Phosphatase; *PTH* Parathyroid Hormone; *25OHD3* 25-Hydroxyvitamin D3; *PSA* Prostate Specific Antigen

Serum normal saline 200 cc/hour was infused, and calcitonin 250 unit every 6 h for 48 h was prescribed; recheck of calcium twice showed 26 mg/dl and 27 mg/dl, so double lumen was inserted and two 2-h hemodialysis sessions were performed. After that, the calcium level decreased, but it was still high (16 mg/dl), so denosumab 60 mg was injected, and serum calcium decreased to 13.5 mg/dl; ultimately, the calcium level gradually decreased to 11.9 mg/dl.

Given the acute kidney damage, abdomen-pelvic sonography was performed; it revealed evidence of multiple mostly hypoechoic structures with foci of calcification, the largest of which was about 72 mm × 62 mm in the right lobe of the liver, suggestive of liver metastasis. Both kidneys were normal in size with severely increased parenchymal echogenicity, suggestive of parenchymal damage. In addition, there were a few stones of up to 6 mm in the lower pole of left kidney. His prostate was enlarged in size at about 30 cc with heterogeneous parenchymal echogenicity.

**Fig. 1:**
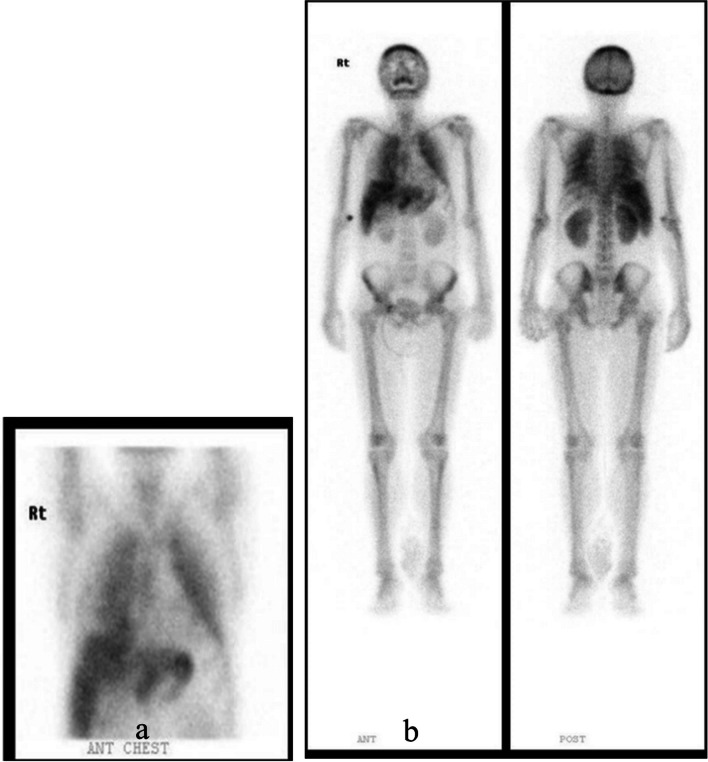
Bone scan (4 h) showed diffuse metastatic calcification in both lungs and stomach (**a**), diffuse heterogeneous uptake in the liver, slight enlargement of both kidneys, and mild degenerative process in the right hip (**b**)

Bone scan showed diffuse metastatic calcification in both lungs and stomach, and diffuse heterogeneous uptake in the liver, which seemed to be a metastatic lesion and mild degenerative process in the right hip (Fig. 1).

Percutaneous biopsy of the liver mass demonstrated a well-differentiated neuroendocrine tumor (low grade) confirmed by immunohistochemistry (IHC) with strongly positive chromogranin and synaptophysin stain, negative CK20, CK7, LCA, and Ki-67 index of < 3%. After consultation with the oncology team, long-acting octreotide 30 mg monthly was suggested. The patient was discharged, but a few days later, he complained from perioral, hand, and feet paresthesia and numbness and developed symptomatic hypocalcemia; his corrected calcium level was 6.5 mg/dl, phosphate level was 2.5 mg/dl, 25 hydroxy vitamin D level was 22.8 ng/ml, and the PTH level was 107 pg/ml. He received intravenous and oral calcium accompanied by 50,000 units of oral ergocalciferol weekly. The symptoms were relieved, and the calcium level was corrected.

**Fig. 2:**
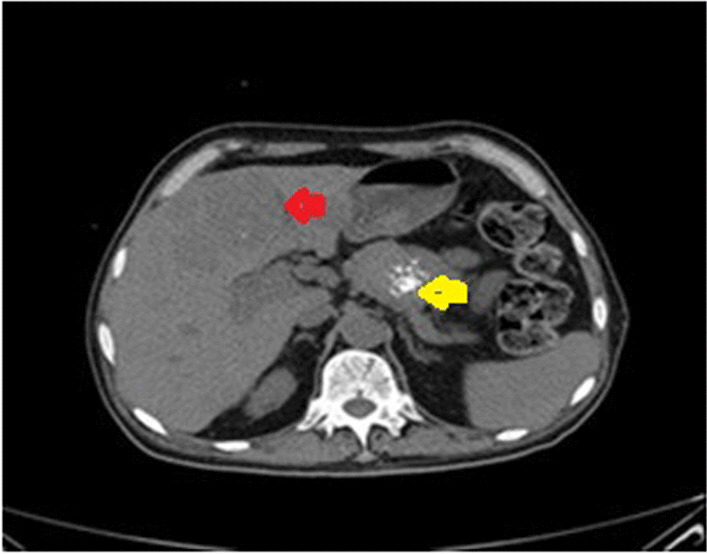
Abdominopelvic CT scan (axial view) without contrast showed a heterogeneous solid mass lesion, which measured 51 mm × 47 mm in the body of the pancreas and contained calcification (yellow arrow). The liver had heterogeneous parenchymal density with multiple varying size hypodense lesions in both lobes; the largest measurement was 73 mm × 67 mm in the left lobe (red arrow) and the foci of calcification in the mentioned lesion can be seen

For further evaluation of the underlying malignancy, computed tomography (CT) scan of the chest, abdomen, and pelvis without contrast was performed; it showed a heterogeneous solid mass lesion, which measured 51 mm × 47 mm in the body of the pancreas containing calcification, suggestive of malignant pancreatic mass lesion. The liver had heterogeneous parenchymal density with multiple varying size hypodense lesions in both lobes; the largest was 73 mm × 67 mm in the left lobe and the foci of calcification in the mentioned lesions were also seen (Fig. 2).

**Fig. 3:**
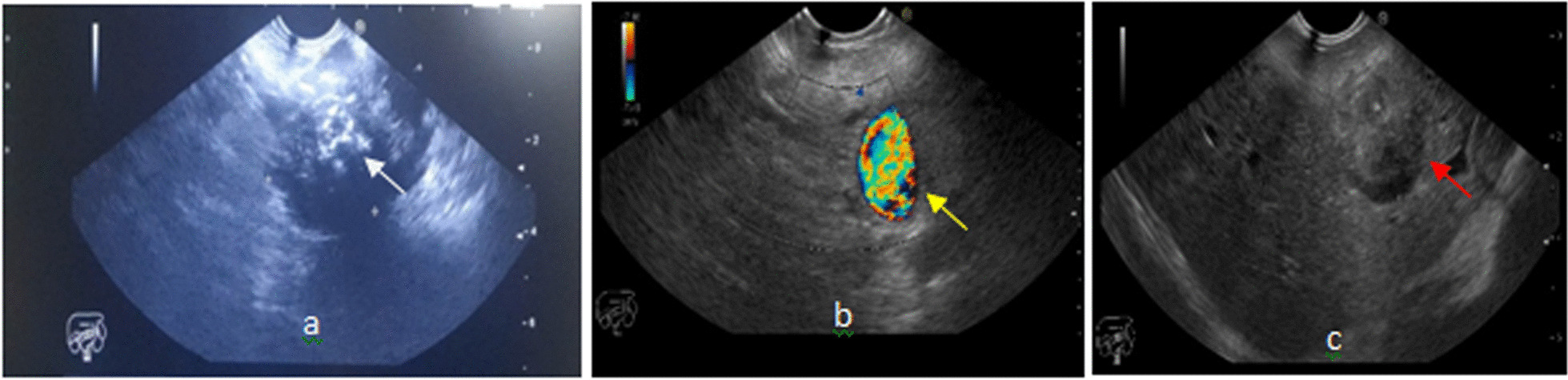
EUS demonstrated a large heterogeneous mass lesion with irregular outline and well demarcation in the body of the pancreas. Diffuse foci of calcification were seen; it was 51 mm × 37 mm (white arrow) (**a**). The lesion had attachment with the common hepatic and proper hepatic artery. The splenic vein was also involved (yellow arrow) (**b**). There were multiple large heterogeneous mass lesions in the left lobe of the liver; the largest one was 53 mm × 51 mm (red arrow) (**c**)

Endoscopic ultrasonography (EUS) was performed for the patient, which demonstrated a large heterogeneous mass lesion with irregular outline and good demarcation in the body of the pancreas. Diffuse foci of calcification were seen; it was 51 mm × 37 mm. The lesion had attachment with the common hepatic artery. Proper hepatic artery seemed to be involved. There were multiple large heterogeneous mass lesions in the left lobe of the liver; the largest one was 53 mm × 51 mm. In addition, the splenic vein was involved (Fig. 3).

**Fig. 4:**
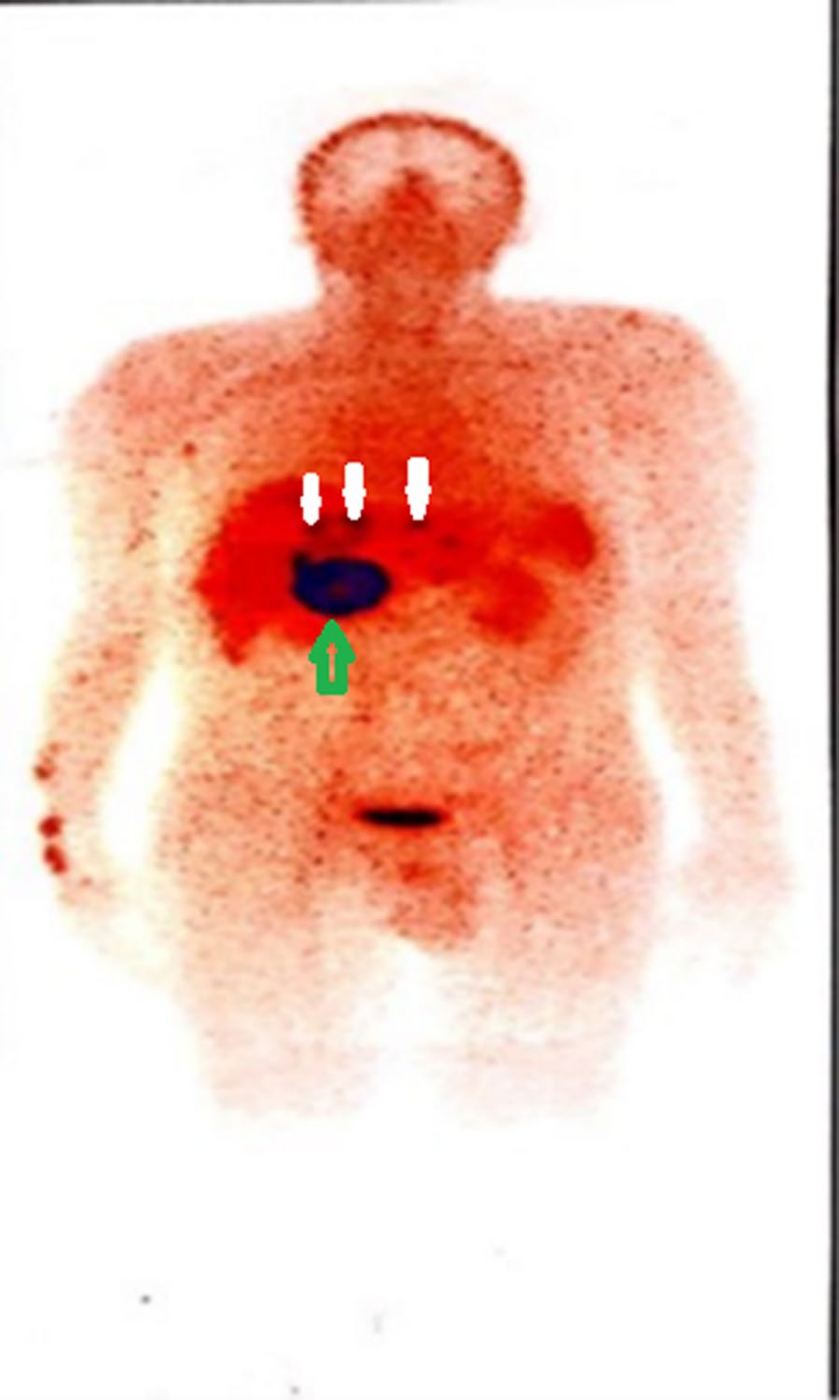
Octreotide scan (anterior view) showed a large donut-shaped octreotide avid lesion in the epigastric region at the right side of the mid-abdomen (green arrow) with multiple metastases in the liver (white arrows)

Octreotide scan revealed a large donut-shaped octreotide avid lesion in the epigastric region at the right side of the mid-abdomen with multiple foci of varying size with abnormally increased radiotracer uptake in the epigastric region and both lobes of the liver (Fig. 4).

Measurement of other probable hormone secretion of NET, such as gastrin and vasoactive intestinal peptide, was not performed because our patient did not have signs and symptoms consistent with these secretions.

The patient was followed by an oncologist and treated with long-acting octreotide 30 mg monthly and everolimus 10 mg daily. He consulted with a transplant surgeon who recommended hemi-pancreatectomy and liver transplantation, but the patient and his family refused this surgical management. Six months later, again he was admitted with nausea, vomiting, and constipation with severe hypercalcemia and acute kidney injury. He had a calcium level of 14.4 mg/dl, phosphate level of 2.7 mg/dl, creatinine level of 2.75 mg/dl, and PTH level of 5 pg/ml. Hydration, medical treatment, and some sessions of hemodialysis were performed for him. After 10 days, he was discharged with calcium of 8.5 mg/dl and creatinine of 1.29 mg/dl.

Now, he has a normal calcium level and is treated with everolimus 10 mg daily and insulin Glargine and Aspart for blood sugar control.

## Discussion

We present a 51-year-old man who complained of nausea, vomiting, and significant weight loss during 1 month before his admission; the laboratory findings revealed severe hypercalcemia accompanied with low PTH. EUS demonstrated a pancreatic mass with advanced vascular invasion and liver metastasis. Pathology findings confirmed pancreatic NET.

Hypercalcemia presents with nausea, vomiting, lethargy, renal failure, and coma. Blood level and duration of hypercalcemia determine the severity of symptoms. Evaluation of hypercalcemia begins with assessment of serum levels of calcium, ionized calcium, PTH, PTHrP, and 1,25-dihydroxyvitamin D. In the setting of paraneoplastic hypercalcemia, laboratory results reveal increased calcium levels, low-to-normal PTH levels, and unusually high PTHrP [5].

Paraneoplastic hypercalcemia due to NET rarely occurs and usually presents in patients with end stage disease [5]. NET can lead to paraneoplastic hypercalcemia through three mechanisms; the most common is PTHrP secretion (85%), the second one is PTH production (11.3%), and the least common one is overproduction of calcitriol (3.8%) [13].

The accuracy of PTHrP assessment has been improved due to the recent double-antibody techniques. Moreover, if PTHrP is high in initial assessment, it can be used as a tumor marker to evaluate the response to treatment. The second mechanism of paraneoplastic hypercalcemia is the ectopic PTH secretion by tumors, most of which are in the lung [14]. Three cases of well-differentiated NET have been reported with paraneoplastic hypercalcemia due to overproduction of calcitriol through the tumor [15–17]. NET, especially PNET, could secrete various peptides, such as calcitonin, vasoactive intestinal peptide, glucagon, gastrin, adrenocorticotropic hormone, and even sometimes multiple peptide cosecretions [18–22].

Elisa *et al*. in an evaluation of 114 patients showed that paraneoplastic hypercalcemia due to NET was relatively more common in males (54.9%) compared with females (45%). In addition, they showed that the most common primary NET histology consisted of pancreatic NET (72.8%), and the second most common was pheochromocytoma (15.8%). The most common site for metastatic disease at paraneoplastic hypercalcemia was the liver (59.1%); the second one was the lymph node (8.2%) and the third was the bone (5.5%). The mean calcium level at presentation was 14 ± 2.7 mg/dl. Hypercalcemia often presented at the onset of the diagnosis; however, progression and relapse of the disease was associated with metachronous hypercalcemia. Moreover, hypercalcemia was often associated with distant metastases, except in 10.5%, where there were just local lymph node metastasis or no metastasis. It seems that it differs for pheochromocytoma, in which the tumor burden at the onset of diagnosis or during progression of the disease determines the hormone secretion capacity by the tumor [13]. Paraneoplastic hypercalcemia usually presents with severe manifestation as an emergency of oncology [5]. However, NET paraneoplastic hypercalcemia, similar to primary hyperparathyroidism, usually has moderate symptoms [13].

Given that primary hyperparathyroidism could be apart from multiple endocrine neoplasia type 1 and type 2, it is important to rule it out before paraneoplastic hypercalcemia syndrome is diagnosed, especially in patients with hypercalcemia simultaneous with NET [23, 24]. Although it is unusual, in patients who present with hypercalcemia due to NET, it would be better to consider ectopic production of PTH whenever high PTH levels are present with no parathyroid-related cause. It is worth recognizing, because it could prevent unnecessary parathyroidectomy or neck exploration [13].

Paraneoplastic hypercalcemia due to PTHrP secretion of NET, in contrast to other malignancies producing PTHrP, is associated with better survival (up to 125 months) [25]. Management of paraneoplastic hypercalcemia is challenging. It is suggested that a multidisciplinary team should control the clinical manifestations and tumor growth. Sometimes control of hypercalcemia requires anti-tumor agents and operative resection of the primary source of the tumor, in addition to the routine treatment of hypercalcemia [26].

In the literature, we found some cases of NET-induced hypercalcemia with variable management strategies. Elisa *et al*. presented a 45-year-old man with hypercalcemia due to pancreatic NET that was treated with distal pancreatectomy and analogous somatostatin. After the operation, due to progression of disease and liver metastasis, everolimus was prescribed for the patient. Then, due to persistent hypercalcemia, management was continued with zoledronic acid and denusumab, which caused jaw osteonecrosis and was stopped. Ultimately, 20 mg prednisolone was administered to the patient, which stabilized the calcium level, but despite the stability of the calcium level, the patient expired [17].

Also, we found another case with well-differentiated bronchial tumor, which presented with lung mass and hypercalcemia of 14.4 mg/dl, PTH of 4.7 pg/ml, and PTHrP of 109 ng/ml that was treated with 4 mg zolendronic acid every 28 days; then, lobectomy was done, which led to normalized calcium level and decreased PTHrP [17].

According to the 2017 World Health Organization (WHO) classification based on ki67 proliferation and mitotic rate, well-differentiated G1–G3 neoplasm, without invasion to the artery of the celiac axis or superior mesenteric artery, is considered as the operable neoplasm. In addition, tumors larger than 2 cm are candidates for surgery; however, tumors between 1–2 cm in low-risk lesions may be considered for surgery rather than surveillance [27]. In the case of pancreatic NETs, the risk of operation rather than the progression or recurrence should be carefully considered [28].

Our patient’s tumor was 51 mm × 37 mm with advanced vascular invasion and liver metastasis and so consensus was hemipancreatectomy and liver transplantation, but the patient and his family refused.

There are several medical treatment plans for symptomatic patients who are not candidates for surgery. Chemotherapy based on temozolomide or capecitabin is considered for functional tumor, but they do not have long-standing effect on hypercalcemia [29–31]. The analog of somatostatin, such as octreotide or lanreotide, is considered for decreased tumor burden, not only in resectable lesion, but also in metastatic lesion, to decrease both symptoms and tumor growth [32–34]. However, this medical treatment sometimes is not sufficient and efficacy decreases over time as result of tachyphylaxis [25].

Our patient’s hypercalcemia was difficult to control because the first presentation was malignant hypercalcemia, and the origin of the tumor was unknown at presentation (we did not have enough time to search for it). He was treated with octreotide 30 mg monthly and everolimus 10 mg daily, but the effect of treatment did not last long and 6 months later, the patient returned with severe hypercalcemia. Moreover, treatment with denosumab in the case of vitamin D deficiency lead to symptomatic hypocalcemia.

Similar to our patient, Noura *et al*. presented a case of malignant hypercalcemia due to high production of PTHrP from pancreatic NET, which resulted in symptomatic hypocalcemia after treatment with denosumab in the case of low-normal vitamin D levels [35].

A recent investigation showed that tyrosine kinase inhibitor drugs, such as sunitinib, could decrease the calcium level in some cases [36, 37].

The limitation of this study was lack of accessibility to assess PTHrP, and 1,25 dihydroxyvitamin D.

## Conclusion

Pancreatic NET could lead to malignant hypercalcemia; before the diagnosis, we should rule out multiple endocrine neoplasia (MEN) type 1. Secretion of PTHrP is the most common cause, and signs and symptoms are usually milder than paraneoplastic syndrome due to hematologic and solid tumor. Generally, survival is better; however, its treatment is challenging, and primary debulking surgery is often required. A team approach to management is important at all points.

## Data Availability

The datasets used and analyzed during the present study are available from the corresponding author on reasonable request.
